# Effects of Therapeutic Hypothermia and Minimal Enteral Nutrition on Short-Term Outcomes in Neonates with Hypoxic–Ischemic Encephalopathy: A 10-Year Experience from Oman

**DOI:** 10.3390/children12010023

**Published:** 2024-12-26

**Authors:** Manoj Malviya, Sathiya Murthi, Dhanya Jayaraj, Vidya Ramdas, Fadia Nazir Malik, Valsala Nair, Nusrabegam Marikkar, Mukesh Talreja, Tariq Sial, Prakash Manikoth, Renjan Varghese, Khalsa Ali Al Ramadhani, Salima Al Aisry, Said Al Kindi, Ahmed Al Habsi, Ranjit Torgalkar, Munawwar Ahmed, Mohammed Al Yahmadi

**Affiliations:** 1 Department of Neonatology, Khoula Hospital, Mina Al Fahal, Muscat 116, Oman; dhanya.jayaraj@moh.gov.om (D.J.); vidhya.ramdas@moh.gov.om (V.R.); fadia.malik@moh.gov.om (F.N.M.); valsala.nair@moh.gov.om (V.N.); nusra.begum@moh.gov.om (N.M.); mukesh.pamandas@moh.gov.om (M.T.); mohammed.sial@moh.gov.om (T.S.); salima.ahmed@moh.gov.om (S.A.A.); mohammed.alyahmadi@moh.gov.om (M.A.Y.); 2 Oman Medical Speciality Board, Statistics, Al-Athaiba, Muscat 130, Oman; sathiya.m@omsb.org; 3 Armed Forces Hospital, Al Khoud, Muscat 112, Oman; prakash.manikoth@mod.gov.om (P.M.); said.alkindi@mcmss.gov.om (S.A.K.); 4 Department of Radiology, Khoula Hospital, Mina Al Fahal, Muscat 116, Oman; renjan.v@moh.gov.om (R.V.); ahmed.alhabsi@moh.gov.om (A.A.H.); munawar.ahmed@moh.gov.om (M.A.); 5 Kentucky Children’s Hospital, University of Kentucky, Lexington, KY 40536, USA; ranjit.torgalkar@uky.edu

**Keywords:** hypoxic–ischemic encephalopathy, magnetic resonance imaging, therapeutic hypothermia, minimal enteral nutrition, MRI scoring system

## Abstract

Background: Therapeutic hypothermia (TH) is the standard treatment for moderate to severe hypoxic–ischemic encephalopathy (HIE) in developed countries, but data on its safety and efficacy in low-middle-income countries are limited and often conflicting. The impact of enteral feeding during TH remains inadequately explored. We aimed to examine TH’s effects on mortality and brain injury and evaluate the safety and effectiveness of minimal enteral feeding during TH. Here, we report our single-center experience with TH over a 10-year period”. Methods: A total of 187 neonates with moderate to severe HIE who underwent cooling were included in this retrospective study. Post-rewarming MRI scans were scored using a validated MRI scoring system. The primary outcomes were mortality and composite outcomes of mortality and brain injury. Results: The mortality rate was 3% in moderate and 25% in severe cases (*p* < 0.001). Overall, 85% (160/187) of neonates received minimal enteral nutrition. Multivariate regression analysis revealed that the severity of HIE at admission (OR 3.4 (1.03–11.6); *p* < 0.04) and gestational age (OR: 0.624 (0.442–0.882); *p* < 0.008) were independent predictors of composite outcomes of death and brain injuries. MRI score was a strong predictor of mortality (AUC: 0.89; *p* < 0.001) and of ability to orally feed at discharge (AUC: 0.73; *p* < 0.001). Conclusions: Mortality rates associated with TH in infants with moderate–severe HIE align with those in high-income countries, and minimal enteral feeding during TH is safe. The severity of HIE, MRI scores, and feeding status are important predictors of outcomes.

## 1. Introduction

Every year, around one million newborns die globally due to intrapartum hypoxic–ischemic events [1]. Hypoxic–ischemic encephalopathy (HIE) is a leading cause of neonatal encephalopathy. It occurs in one to five out of every 1000 live births in developed countries and 5 to 20 times more often in developing countries [2,3]. Between 10 and 60% of moderate to severe HIE cases result in death, and 25% of survivors experience abnormal neurodevelopmental outcomes [3,4].

For over a decade, therapeutic hypothermia has been the standard treatment for moderate/severe HIE in infants over 35 weeks of gestational age in developed countries [5,6]. A Cochrane review by Jacob et al., incorporating 11 studies performed in developed countries, showed that therapeutic hypothermia is safe and reduced combined outcomes of death and major neurodevelopmental disability by 25% at 18 months of age [3]. However, data from low- and middle-income countries regarding the safety and efficacy of therapeutic hypothermia are conflicting [7,8]. A recent large randomized study conducted in three low- and middle-income countries (LMICs) revealed that therapeutic hypothermia is harmful, increasing mortality at hospital discharge (36% vs. 24%, *p* < 0.008) and at 18 months of age (42% vs. 31%) [9]. Conversely, a systemic review of 28 randomized controlled trials showed reduced mortality with therapeutic hypothermia in LMICs and high-income countries (HICs). The maximum reduction was observed in LMICs compared to HICs (50% vs. 24%) [10]. We found no randomized trials examining the safety and efficacy of therapeutic hypothermia from Middle Eastern countries. The severity of brain injuries as observed by MRI after therapeutic hypothermia predicts long-term neurodevelopment; however, its role in predicting short-term outcomes, particularly mortality, is not clearly defined.

Although therapeutic hypothermia for HIE is well studied, there is scant research on other aspects of the treatment of neonates with HIE, such as medication therapy, the need for mechanical ventilation, and nutrition, specifically enteral nutrition. Enteral milk feeding was withheld during therapeutic hypothermia in the trials that established therapeutic hypothermia as the standard of care for HIE [3]. Review articles and guidelines for HIE do not make specific recommendations about enteral nutrition during therapeutic hypothermia [11,12], but it is a universal practice to withhold feedings during therapeutic hypothermia to avoid necrotizing enterocolitis due to mesenteric hypoperfusion [13,14].

A literature search we performed identified 112 articles related to enteral nutrition in neonates with HIE, and no randomized controlled trials were found on this topic. In our center, we have been feeding infants enterally with mother’s milk since we started using therapeutic hypothermia as the standard treatment for HIE in 2013. Moreover, data are emerging that mother’s milk may be safe and potentially beneficial during hypothermia in modulating the inflammatory response [15].

Given the limited information available about the safety and efficacy of therapeutic hypothermia in this region, it is necessary to investigate its safety in the Omani population, especially after the HELIX trial raised concerns that therapeutic hypothermia may not be safe for all populations [8]. This study aimed to investigate the clinical profile and short-term outcomes of infants who received therapeutic hypothermia, including its effect on the mortality and severity of brain injuries as observed on MRI. This study also examined the safety of enteral feeding during therapeutic hypothermia.

## 2. Methods

### 2.1. Study Design, Settings, and Participants

This paper presents a single-center observational retrospective cohort study conducted at Khoula Hospital in Oman. The NICU is a 51-bed level III/IV tertiary care unit with 5000 deliveries and 1200–1300 neonatal admissions per annum, which is served by seven neonatal consultants and 105 clinical nurses. The inclusion criteria were neonates with moderate to severe HIE, inborn or outborn, who were admitted to the NICU within 6 h of life and received therapeutic hypothermia between 1 January 2013 and 31 December 2023. Neonates were selected for cooling by applying stringent eligibility criteria based on criteria used in a landmark study by the National Institute of Child Health and Human Development (NICHD) (Table 1) [16]. EEG-based criterion C was introduced as an optional criterion in 2021 when criterion B was not fulfilled (Table 1). Therapeutic hypothermia was provided via a servo-controlled whole-body cooling blanket (Tecotherm Neo; Inspiration Health Care, Crawley, UK), which kept the baby’s temperature at 33.5 °C for 72 h using a rectal probe. TH was followed by an automated, gradual rewarming process at a rate of 0.5 °C per hour to reach normal temperature.

The severity of encephalopathy was categorized into mild, moderate, and severe based on modified Sarnat staging [3,17]. Supportive intensive care, including sedation, inotropes, blood products, and anticonvulsants, were used at the treating neonatologist’s discretion. However, certain care practices were standardized. For sedation, morphine infusion was initiated at the onset of therapeutic hypothermia. Phenobarbitone was the first choice of medication for the treatment of seizures, which was followed by phenytoin and midazolam infusion for refractory seizures.

Minimal enteral feeding with mothers’ exclusive breast milk was initiated during therapeutic hypothermia as soon as it was available and progressed as tolerated. The baby was kept nil per oral if it was not available. Donor breast milk was not administered due to religious preference. Neonates were fed every 4–6 h on day one and every 3–4 h thereafter during therapeutic hypothermia. Demand feeding was more common post-rewarming. Inotropic support was provided based on clinical and ECHO findings. Volume-targeted ventilatory support was used if the baby required mechanical ventilation.

All neonates who survived therapeutic hypothermia underwent MRI imaging within one week of therapeutic hypothermia, typically within 5–8 days. Three radiologists re-examined all the MRI scans and classified the pattern of injuries according to the validated Washington University of Neonatal Neurology and Physiology Research laboratory MRI scoring system [18]. A cumulative score of MRI abnormal signal was determined by adding up the five regional subscores (subcortical + white matter + cortical + cerebellar +brainstem).

The final MRI score ranges from 0 to 138 and is categorized as follows: no injury (0), mild (1–11), moderate (12–32), and severe (33–138). An MRI score cut-off of 10.5–11.5 significantly predicts outcomes. Disagreement was resolved through consensus.

The exclusion criteria were neonates < 35 weeks of gestational age, major malformations, congenital infections, or inborn errors of metabolism and death in delivery rooms. Hypothermia was terminated if the neonate showed severe primary pulmonary hypertension, refractory hypotension despite optimal inotropic and volume support, or a life-threatening or massive hemorrhage. As per hospital policy and religious preferences, the withdrawal of life-sustaining therapy was not performed during therapeutic hypothermia. When the clinical condition and MRI report indicated a very poor long-term prognosis, and if the family consented, a shared decision “not to escalate life-sustaining measures” was implemented.

### 2.2. Data Collection

Clinical and outcomes data for all infants who underwent therapeutic hypothermia were retrieved from the hospital database.

### 2.3. Outcomes

The two primary outcomes were the effect of therapeutic hypothermia on mortality before discharge and the incidence of mortality and/or any grade of brain injuries on MRI.

Secondary outcomes: Feeding-related outcomes include the day of initiation and reaching full feed, feeding at discharge (oral feeds vs. NGT), and the incidence of necrotizing enterocolitis. Other secondary outcomes were the duration of mechanical ventilation in days, the need for inotropic support in the first 48 h of life, PPHN requiring the use of iNO, the need for seizure medication at discharge, acute kidney injury measured by creatinine levels and MRI scores based on severity of encephalopathy and survival status.

### 2.4. Ethics Approval

Parental consent was not obtained as all data were anonymized. Ethical approval was obtained from the hospital’s ethical committee. (MOH/DGKH/REC/24/29118).

### 2.5. Statistical Analysis

Clinical and outcome data of neonates receiving therapeutic hypothermia were compared in each category of HIE severity. Categorical variables and continuous variables are presented as n (%) and median (IQR) or mean (±SD), respectively. The Mann–Whitney U test was used to analyze continuous data, and categorical variables were compared using the Chi-square test for among-group analysis. Multivariate logistic regression analyses were performed to identify significant risk factors associated with hospital mortality at discharge and/or brain injuries on MRI. *p*-values of <0.05 were considered statistically significant; all tests were two-sided. The predictive performance of Sarnat stages, cord blood gas, and the need for resuscitation on MRI score, as well as the predictive value of MRI scores on death or feeding status at discharge, were assessed using the area under the receiver operating characteristic curve. The statistical package SPSS 29.0 was used for the analysis.

## 3. Results

### 3.1. Birth Characteristics and Severity of Encephalopathy at Admission

The clinical characteristics of infants with moderate HIE and severe HIE at admission are described in Table 2. Infants with severe HIE were more likely to be outborn, and they also had lower Apgar scores, lower cord gas pH, a higher cord gas base deficit, and a greater proportion of required resuscitation.

#### Therapeutic Hypothermia and Multiorgan Outcomes

Table 3 describes the multiorgan outcomes of infants with moderate and severe HIE who received TH. Infants with severe HIE had a higher incidence of seizures at admission and a greater need for mechanical ventilation, blood transfusion, and nitric oxide. They were more likely to die before discharge or go home on seizure medication.

### 3.2. Birth Characteristics of Survivors vs. Non-Survivors of HIE Who Were Treated with TH

A total of 187 neonates received therapeutic hypothermia during the study period. Overall, 88% (165/187) survived and were discharged home, while 12% (22/187) died before discharge (Table 4). Infants who died compared to those who survived were of lower birth weight, had low Apgar scores, were more likely to require resuscitation, and had higher Thompson neuro scores.

#### Multiorgan Outcomes of Survivors vs. Non-Survivors of HIE Who Were Treated with TH

Infants who died, compared to those who survived, were more likely to require blood transfusions, inotropic support, mechanical ventilation, and nitric oxide therapy and more likely to be discharged with seizure medication (Table 5).

### 3.3. Feeding Outcomes

A total of 160/187 (85%) neonates received minimal enteral nutrition with exclusive breastmilk feeding (BMF) via nasogastric tube during therapeutic hypothermia treatment. Of these, 16 neonates received the initiation of feeding on day 1, 120 neonates on day 2, and 24 neonates on day 3. Neonates with severe encephalopathy compared to moderate encephalopathy had delayed milk feeding initiation. At discharge, they were less likely to exclusively breastfeed and more likely to use nasogastric tube feeding (NGT) (Table 6). Only one baby developed medical necrotizing enterocolitis (NEC) after receiving formula milk on day four, and this baby had severe encephalopathy.

### 3.4. MRI and Clinical Outcomes

MRI was available in 90% (169/187) of infants to assess the severity of brain injury with 81% (137/169) having it performed before eight days of life. Eight patients died before the MRI could be performed. When combined, 37% (69/187) had no obvious brain injury and were alive, and 63% (118/187) had some form of brain injury or died. MRI scores increased significantly with the severity of encephalopathy, indicating a progressive involvement of subcortical structures, white matter, cortical areas, the cerebellum, and the brainstem (Table 7). MRI scores were significantly higher for infants who died vs. those discharged home for severe vs. moderate Sarnat stage HIE (Table 8) and for those infants receiving mixed NGT/oral feeding vs. all breastfeeding infants at discharge [median (IQR): 16 (2–46) vs. 0 (0–11), *p* < 0.001].

When looking at the MRI injury score’s prediction performance, it was found to be a good predictor of mortality (AUC: 0.89; *p* < 0.001), a fair predictor of the need for seizure medication at discharge (AUC: 0.78; *p* < 0.001), and a fair predictor of feeding status (breastfeeding at discharge) (AUC: 0.73; *p* < 0.001) with optimal predictive thresholds of 11, 12 and 10, respectively.

## 4. Discussion

This study outlines the care practices and outcomes of infants with moderate to severe HIE who underwent therapeutic hypothermia at a single center in Oman over the past ten years. It is the first study from Oman and the Middle East on neonates with HIE who received therapeutic hypothermia.

After the HELIX study, a multicenter randomized trial, concerns about therapeutic hypothermia’s safety and efficacy emerged in LMICs [9]. The study found a higher discharge mortality rate (36% vs. 24%) with hypothermia, leading authors to recommend normothermia as standard care in LMICs despite available hypothermia treatment in NICUs [19]. Our results differed from HELIX, aligning more with high-income countries’ data, showing a total discharge mortality rate of 12% with moderate HIE at 2.5% and severe HIE at 24.7%. This is less than half of HELIX’s rate and comparable to HICs’ published data [5,6,16,20].

The reported mortality associated with therapeutic hypothermia for HIE varies with HIE severity. Low rates are found in studies that recruited more neonates with mild to moderate HIE compared to severe cases [6,20]. Turkish and Canadian studies (24 NICUs) reported mortality rates of 6% and 5–17% with a higher number of mild–moderate cases. A Canadian unit recruiting more neonates with mild HIE (37%) had the lowest mortality of 5%, while one with fewer mild cases (13%) had the highest at 17% [20]. Similarly, in Turkey, 82% of neonates had mild–moderate HIE with a 2.3% mortality, while 18% with severe HIE had a 29% rate [6]. A review of 12 studies before the therapeutic hypothermia era found higher mortality in Sarnat stage III HIE than stage II (67% vs. 6%) [21]. We speculate that the high mortality in the HELIX trial likely stems from selection bias, as it recruited more neonates with severe HIE and presented combined mortality data without distinguishing between moderate and severe HIE [9]. Additionally, the patient selection process for therapeutic hypothermia is not robust, lacks standardization, and is prone to bias. It is a two-step process that involves assessing evidence for hypoxia ischemia and moderate to severe encephalopathy [3,11]. The Apgar score for hypoxic ischemia can be subjective, while diagnosing encephalopathy through neurological exams is challenging, subjective, and affected by maternal anesthesia or neonatal sedation [17].

Evidence shows that multiorgan dysfunction in infants with HIE early in life impairs cerebral blood flow regulation, exacerbates secondary energy failure, and may lead to mortality and long-term morbidities [12]. Our findings support previous research showing that multiorgan dysfunction severity correlates with HIE severity and increases mortality risk [22,23]. Studies show that hypotension increases the risk of death and brain injuries in neonates receiving TH [24,25]. However, like in our study, treatment with inotropes does not affect these outcomes [25]. A study on the impact of seizure burden on neurodevelopment at 18 months found a high seizure burden and multiple antiepileptic drugs linked to poor outcomes [26]. In this cohort, 70% of infants had seizures, and 28% were discharged on one medication with increased risks of adverse events (death or brain injury on MRI). We found no differences in primary outcomes between inborn and outborn neonates, timing of cooling initiation, or antenatal events. Therapeutic hypothermia should begin within 6 h of birth. Two recent observational studies, like ours, also found no differences in mortality and MRI injuries when initiated before 3 h of age [6,27].

Feeding practices during therapeutic hypothermia vary widely [3,14,28]. A Canadian study found that only 8–42% of neonates were fed in the first 48 h [20]. In our study, 73% of neonates during therapeutic hypothermia were exposed to breast milk within 48 h, and 45% were exclusively breastfeeding at discharge. Similarly to our cohort, studies in Sweden and the UK showed that feeding during therapeutic hypothermia led to more breastfeeding at discharge [28,29]. The main reason to avoid feeding babies during therapeutic hypothermia is the risk of necrotizing enterocolitis (NEC). In our cohort, only one baby developed NEC after formula feeding post-TH. A UK study of 6030 babies found that 31.1% were fed during TH with only 0.1% developing NEC [29]. Evidence is growing that feeding during therapeutic hypothermia is safe and beneficial. However, prospective control studies are needed to examine optimal nutritional support, including milk type, timing, frequency, and volume during hypothermia and rewarming, and its impact on outcomes.

MRI within the first eight days of life is a key tool for assessing injury extent, timing, short-term mortality, and long-term neurodevelopment [30]. Our study showed that 65% (110/169) of infants had MRI abnormalities with 41% (70/169) having moderate to severe issues. In contrast, Jacob et al. found 51% (195/384) of neonates receiving therapeutic hypothermia exhibited moderate to severe abnormalities. The high abnormality rate in our study may result from using DWI along with T1 scans compared to the systemic review. Additionally, 81% of MRIs were conducted before eight days of life versus 15 ± 12 days in the review, leading to pseudo-normalization [18,31]. This study shows that the MRI scoring system predicts discharge outcomes. Neonates with higher MRI scores had a greater likelihood of death (AUC: 0.89; *p* < 0.001) and were discharged on nasogastric tube feeding (AUC:0.73; *p* < 0.01). In contrast, those with lower or normal MRI scores were more likely to achieve full feeding earlier and to breastfeed at discharge (AUC: 0.73; *p* < 0.001). MRI scores provide a clear, objective framework for clinicians and parents, predict outcomes, and facilitate counseling.

The study’s strength is its large sample size with 90% of patients having MRI scans examined by a validated MRI scoring system and standardized selection criteria for therapeutic hypothermia. This 10-year study establishes that feeding during hypothermia is safe and may positively impact short-term outcomes, including a lower mortality rate versus published reports. Our findings are relevant to infants with moderate to severe hypoxic–ischemic encephalopathy treated per a published protocol. Limitations include the retrospective design, lack of a control group, and absence of long-term neurodevelopmental outcome data. Although neonates received hypothermia per a strict protocol, we did not assess individual cases for context or compliance.

## 5. Conclusions

This study indicates that the mortality rate from therapeutic hypothermia in infants with moderate to severe HIE matches that of high-income countries but is better than earlier control studies that validated therapeutic hypothermia as standard therapy. Feeding during therapeutic hypothermia is safe, and urgent prospective studies are needed to investigate optimal nutrition during therapeutic hypothermia. The severity of HIE at admission and MRI scores post-therapeutic hypothermia are significant predictors of mortality and feeding status at discharge.

## Figures and Tables

**Table 1:** Eligibility criteria for therapeutic hypothermia.

Qualifying Criteria (All is a must) Gestational age is 35 + 0 weeks or greater Birth weight ≥ 1800 g Age in hours is less than 6 h	
Criteria A (at least one) Apgar scores less than 5 at 10 min Base deficit is equal to or greater than 16 Cord pH is less than or equal to 7 in arterial, venous or capillary blood gas within 60 min after birth Requirement for positive ventilation at 10 min	Criteria B (Either one of following two) Evidence of moderate-to-severe encephalopathy demonstrated by the presence of seizures OR Neonate shows changes in any three of the following Decreased level of consciousness Decreased or no spontaneous activity Abnormal posture Abnormal tone Weakness in any primitive reflexes Constricted or variable pupils Bradycardia or variable heart rate Shallow breathing or apnea
Criteria C (not mandatory) Only to be considered if “Criteria B” is not satisfied Any of the following integrated EEG findings Normal background with electrical seizure activity Moderately abnormal background activity (<5 μV and >10 μV) Suppressed background activity (<5 μV and <10 μV) Continuous seizure activity	

**Table 2:** Birth characteristics of infants with moderate HIE and severe HIE at admission.

	Moderate HIE (n = 110)	Severe HIE (n = 77)	*p*-Value
GA (week), mean (SD)	38.75 (1.54)	38.62 (1.56)	0.570
Birth weight (gm), mean (SD)	3130.7 (460.3)	3016.7 (517.3)	0.115
Gender (Male), n, (%)	70 (63)	50 (64)	0.878
Inborn, n, (%)	55 (50)	24 (31)	0.011
Resuscitation ^a^ n (%)	17 (15)	40 (52)	0.001
APGAR at 5-min, median IQR	5 (4–6)	4 (3–5)	0.001
First gas pH, mean (SD)	6.97 (0.17)	6.88 (0.20)	0.002
First gas BE, mean (SD)	−16.06 (5.0)	−19.68 (5.6)	0.001

HIE = hypoxic–ischemic encephalopathy, GA = gestational age, ^a^ = chest compression and/or adrenaline, SD = standard deviation, BE = base excess.

**Table 3:** Multiorgan outcomes of infants with moderate and severe HIE who received TH.

	Moderate HIE (n = 110)	Severe HIE (n = 77)	*p*-Value
Seizure n (%)	84 (76)	71 (92)	0.005
Seizure medication at discharge, n (%)	19 (17)	34 (47)	0.001
Inotropes used in <48 h, n (%)	43 (39)	37 (48)	0.23
Blood transfusion, n (%)	12 (11)	21 (27)	0.006
PPHN iNO use, n (%)	4 (4)	9 (12)	0.04
Mechanical ventilation days, mean (SD)	2.10 (2.98)	8.39 (13.08)	0.001
Creatinine (µmol/L) at 24 h, mean, (SD)	67.60 (31.4)	77.49 (33.0)	0.006
Died, n (%)	3 (3)	19 (25)	0.001

TH = therapeutic hypothermia, HIE = hypoxic–ischemic encephalopathy, PPHN = persistent pulmonary hypertension. iNO = nitric oxide, SD = standard deviation.

**Table 4:** Birth characteristics of survivors compared to non-survivors of HIE who received treatment with TH.

	Alive (n = 165)	Dead (n = 22)	*p*-Value
Birth characteristics			
GA (wk), mean (SD)	38.7 (1.5)	38.6 (1.1)	0.88
Birth weight (gm), mean (SD)	3110.98 (482.6)	2880.32 (478.2)	0.036
Gender (Male), n, (%)	109 (66)	11 (50)	0.156
NVD, n, (%)	63 (39)	7 (32)	0.828
Inborn, n, (%)	69 (83)	10 (13)	0.82
Resuscitation ^a^ n (%)	44 (27)	13 (59)	0.005
APGAR at 5 min mean (SD)	4.94 (1.90)	3.38 (1.68)	0.001
First gas pH, mean (SD)	6.94 (0.17)	6.86 (0.28)	0.23
First gas BE, mean (SD)	−17.1 (5.1)	−20.0 (7.6)	0.044
Severity of HIE according to Thompson neuro-score, n = 140			
<10 n (%)	80 (62.0)	1 (9.1)	0.001
11–14	27 (21)	3 (27)	
>15	22 (17)	7 (64)	

HIE = hypoxic–ischemic encephalopathy, TH = therapeutic hypothermia, GA = gestational age, NVD = normal vaginal delivery, ^a^ = chest compression and/or adrenaline, SD = standard deviation, BE = base excess.

**Table 5:** Multiorgan outcomes of survivors vs. non-survivors of HIE who were treated with TH.

	Alive (n = 165)	Dead (n = 22)	*p*-Value
Seizure n (%)	136 (82)	19 (86)	0.772
Seizure medication at discharge, n (%)	42 (26)	13 (55.0)	0.016
Inotropes used in < 48 h, n (%)	64 (39)	16 (73)	0.005
Blood transfusion, n (%)	20 (12)	13 (59)	0.001
PPHN iNO use, n (%)	9 (5)	4 (19)	0.04
Mechanical ventilation days, mean (SD)	3.3 (7.0)	14.8 (15.7)	0.000
Creatinine (µmol/L) at 24 h Mean, (SD)	69.9 (30.5)	84.1 (42.0)	0.16

HIE = hypoxic–ischemic encephalopathy, TH = therapeutic hypothermia, PPHN = persistent pulmonary hypertension, iNO = nitric oxide, SD = standard deviation.

**Table 6:** Feeding outcomes based on the severity of encephalopathy.

Clinical Characteristics of the Study Patients	Moderate HIE (n = 110)	Severe HIE (n = 77)	*p*-Value
Day initiation median IQR	2 (2–3)	2 (2–4)	0.007
Day reached full feed median (IQR)	5 (4–7)	6 (5–8)	0.001
Feeding status at discharge			
All breastfeeding at discharge, n (%)	66 (62)	19 (25)	0.001
BF plus oral at discharge, n (%)	35 (32)	26 (34)	
NGT at discharge, n (%)	5 (4)	13 (17)	

**Table 7:** MRI scores and severity of HIE.

	Moderate HIE (n = 110)	Severe HIE (n = 77)	*p*-Value
MRI score, median IQR	1 (0–12)	28 (4–57)	0.001
Normal n (%)	50 (49.5)	9 (13)	0.001
Mild, n (%)	25 (24.8)	15 (22)	
Moderate, n (%)	21 (20.8)	15 (22)	
Severe, n (%)	5 (5.0)	29 (43)	

**Table 8:** MRI scores of infants who survived and those who did not after TH treatment.

	Alive (n = 165)	Dead (n = 22)	*p*-Value
MRI scores, median IQR	3 (0,22),	62 (29,11)	0.001
Normal n (%)	59 (38.1)	0	0.001
Mild, n (%)	40 (26)	0	
Moderate, n (%)	32 (21)	4 (18	
Severe, n (%)	24 (15)	10 (71)	

## Data Availability

The datasets generated and analyzed during the current study are available from the corresponding author upon reasonable request due to privacy.
